# Effect of various physical parameters on surface and build-up dose for 15-MV X-rays

**DOI:** 10.4103/0971-6203.71761

**Published:** 2010

**Authors:** Girigesh Yadav, R. S. Yadav, Alok Kumar

**Affiliations:** Department of Physics, D.A.V. (P.G.) College, Kanpur, U.P, India; 1 Department of Medical Physics, AMRI Hospitals, Kolkata, India

**Keywords:** Focus-to-skin distance, motorized 60° wedge, percentage depth dose at surface, Perspex block tray, surface dose

## Abstract

The purpose of this study was to find out the effect of various physical parameters on the skin and build-up doses of 15-MV photon beams. The effects of field dimensions, acrylic shadow tray, focus to-skin distance (FSD) on surface and buildup dose were determined for open, motorized 60° wedge (MW) and blocked fields. A ‘Markus’ plane parallel plate chamber was used for these measurements in an Elekta (6–15MV) linear accelerator. The surface dose for MW fields was lower than the dose for an open field, but the trend reversed for large fields and higher degree wedges. With the use of an acrylic shadow tray, the surface dose increased for all field sizes, but the increase was dominant for large fields. The surface dose for blocked fields was lower than the dose for open fields. The percentage depth dose of 10 × 10 cm^2^ field at surface (PDD_0_) for open beam were 13.89%, 11.71%, and 10.74% at 80 cm, 100 cm, and 120 cm FSD, respectively. The blocking tray increased PDD_0_ of 10 × 10 cm^2^ field to 26.29%, 14.01%, and 11.53%, while the motorized 60° wedge decreased PDD_0_ to 11.32%, 9.7%, and 8.9 % at these FSDs. The maximum PDD difference seen at surface (i.e., skin) for 5 × 5 cm^2^, 15 × 15 cm^2^, and 30 × 30 cm^2^ are 0.5%, 4.6%, and 5.6% for open field and 0.9%, 4.7%, and 7.2% for motorized 60° wedge field, when FSDs varied from 80 cm to 120 cm. The maximum PDD difference seen at surface for 5 × 5 cm^2^, 15 × 15 cm^2^, and 30 × 30 cm^2^ fields are 5.6%, 22.8%, and 29.6%, respectively, for a 1.0-cm perspex-blocking tray as the FSD is changed. The maximum PDD difference was seen at the surface (i.e., skin) and this decreased with increasing depth.

## Introduction

In radiotherapy, high-energy medical linear accelerators are used for the treatment of cancer. Megavoltage X-ray beams are used for the treatment of deep-seated tumors. Megavoltage X-rays have a skin-sparing effect, whereby a higher dose is deposited in the deep tissues than in the skin.[1] This effect is due to longitudinal disequilibria of electrons excited by the high-energy X-rays. Ideally, the dose at the surface should be negligible; however, this is never achieved because there are two sources of contamination: one is treatment head materials[2,3] and the other is treatment setup parameters[4,5] such as the field size, the use of beam modifying devices, and the focus-to-surface distance (FSD). With isocentric tumor treatment (i.e., when the tumor is located at the isocenter), the FSD can vary significantly. This effect changes the parameters of clinical treatment and the distance of the skin from the electron contamination–producing devices located in and near the head of the medical linear accelerator. The amount of these contaminant electrons and low-energy photons will affect surface and build-up regions dose.[6] Knowledge of how different parameters affect the surface and build-up region dose is essential for proper treatment. Excessive radiation dose to the patient’s skin can cause early radiation effects such as erythema or late effects such as hypoxia, fibrosis, etc.

We performed a comprehensive set of surface and build-up dose measurement on the precise linac to examine effects of field size (FS) motorized 60° wedge (MW), acrylic block tray, and focus-to-surface distance (FSD).

## Materials and Methods

Surface and build-up region dose measurements were carried out for 15-MV photons, for various field sizes, with beam modifiers at different FSDs. The Elekta precise linear accelerator (Elekta Oncology Systems, Crawley, UK) in our clinic delivers 6- and 15-MV photons and six-electron beams; it has a multileaf collimator (MLC) having 40 pairs of leaves, with each leaf projecting 1.0 cm width at the isocenter. Measurements were carried out with a Markus-type parallel-plate ion chamber (0.055-cc measuring volume, 0.03-mm wall thickness, acrylic electrode, graphite-coated, 5.3 mm in diameter, 2.0-mm electrode separation, and a 0.2-mm guard ring) with a PTW electrometer (PTW Freiburg, Germany). The chamber was embedded in an acrylic slab phantom. The outer dimension of the phantom was 300 mm × 300 mm, with 1.0-mm to 300-mm thickness. To overcome the perturbation of the measured dose on surface and build-up regions using a plane parallel ion chamber with fixed plate separation an over response correction factor was applied for the Markus chamber.[7] A polarizing potential of +300 V was reversed for all measurements because of a large polarity effect observed at the phantom–air interface.[8]

The percentage build-up region depth dose data (depths ranging from 0 to 4 cm) were measured for open, motorized wedge, and tray fields for different field sizes (3 × 3 cm^2^, 5 × 5 cm^2^, 10 × 10 cm^2^, 15 × 15 cm^2^, 20 × 20 cm^2^, 25 × 25 cm^2^, and 30 × 30 cm^2^) at 80, 100, and 120 cm FSDs. Readings at the phantom surface (depth = 0) were normalized to readings at the depth of dose maximum for the individual field setup (because depth of dose maximum was not constant for all the setups – it changes with FSD, field size, and beam modifiers) to obtain relative surface and build-up doses. Percentage depth dose at surface (PDD_0_) is defined as:

$$\mathrm{PDD}\;=\;\frac{\mathrm{MR}\;\mathrm{at}\;a\;\mathrm{depth}}{\mathrm{MR}\;\mathrm{at}\;\mathrm{their}\;\mathrm{respective}\;d\;\max\;}\;\times \;100-O.C.F.$$

$${\mathrm{PDD}}_{0}\;=\;\frac{\mathrm{MR}\;\mathrm{at}\;\mathrm{zero}\;\mathrm{depth}}{\mathrm{MR}\;\mathrm{at}\;\mathrm{their}\;\mathrm{respective}\;d\;\max\;}\;\times \;100-O.C.F.$$

Where MR is the dosimeter reading and O.C.F is a chamber over response correction factor.

**Figure 1:**
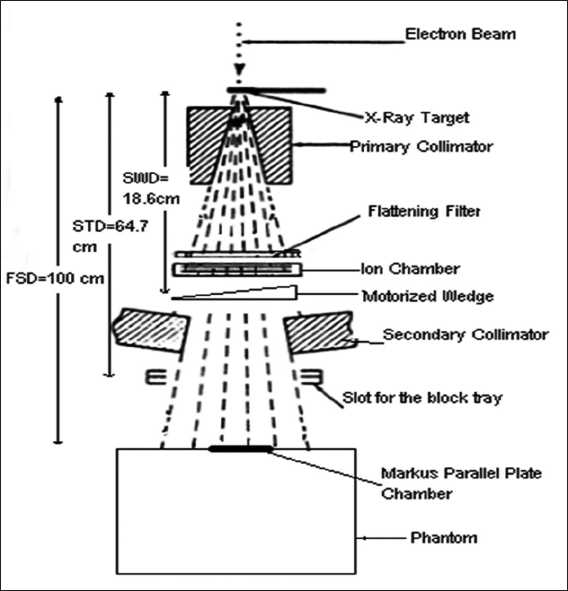
Experimental setup

An acrylic block tray of 10-mm thickness was placed in the beam to determine its effect on the surface and build-up dose. The tray is normally in use during treatments to support the cerrobend blocks and it is placed at the accessory tray holder located at a distance of 64.7 cm from the source. The effect of a motorized 60° wedge on the surface and build-up dose was measured by inserting the motorized wedge (located at 18.6 cm from the source) in the beam. The collimator setting at 100 cm FSD defined the field sizes. The experimental setup is shown in Figure 1.

## Results

### Surface dose characteristics

Table 1 shows the percentage depth dose at surface (PDD_0_) for open, motorized 60° wedge (MW), and tray fields at various FSDs for different field sizes. It was found that the values of PDD_0_ increase as field size increases. The percentage depth dose at surface (PDD_0_) for open beams, at 80 cm, 100 cm, and 120 cm FSDs were 13.89%, 11.71%, and 10.74%, respectively, for a 10 × 10 cm^2^ field. The blocking tray increased PDD_0_ to 26.29%, 14.01%, and 11.53%, while the motorized wedge decreased PDD_0_ to 11.32%, 9.74%, and 8.9% for the 80 cm, 100 cm, and 120cm FSDs, respectively.

**Table 1:** Percentage depth dose at surface for open, wedge, and tray fields at 80 cm, 100 cm, and 120 cm FSD for different field sizes

*Field Size (cm × cm)*	*PDD_0_ for open fields*			*PDD_0_ for MW fields*			*PDD_0_ for tray fields*		
	
	*FSD = 80 cm*	*FSD = 100 cm*	*FSD = 120 cm*	*FSD=80 cm*	*FSD = 100 cm*	*FSD = 120 cm*	*FSD = 80 cm*	*FSD = 100 cm*	*FSD = 120 cm*
3 × 3	3.45	3.20	3.15	2.05	1.71	1.31	5.55	3.31	3.07
5 × 5	5.77	5.30	5.27	3.98	3.91	3.09	10.76	5.88	5.2
10 × 10	13.89	11.71	10.74	11.32	9.74	8.9	26.29	14.01	11.53
15 × 15	21.7	18.31	17.11	19.33	15.45	14.62	41.05	22.96	18.3
20 × 20	29.15	24.94	23.4	28.13	23.3	21.78	53.23	32.17	25.43
25 × 25	34.56	29.93	27.04	35.03	28.97	25.61	63.09	40.49	32.28
30 × 30	39.06	34.47	33.4	41.37	36.82	34.20	71.33	47.69	41.7

IPD_0_= Percentage depth dose at surface

### Dependence of field size

The percentage depth dose at surface (PDD_0_) increased almost linearly with field size (~1.124%/cm), (~1.643%/cm), and (~1.20%/cm) for open, tray, and wedge field, respectively, at 100 cm FSD [Table 1].

### Dependence of FSD

The percentage depth dose at surface (PDD_0_) of tray fields decreased rapidly with increasing FSD tray field, but for the open and wedge field it decreased slightly with increasing FSD [Table 1].

### Buildup data

Figures 2a and 2b show the build-up curves for a 5 × 5 cm^2^, 15 × 15 cm^2^, and 30 × 30cm^2^ field (as at 100 cm FSD), at 80 cm, 100 cm, and 120 cm FSD for open and motorized 60° wedge fields. It can be seen that percentage dose build-up does not vary significantly with FSD for open and motorized wedge fields. The maximum PDD difference seen at the surface (PDD_0_) for 5 × 5cm^2^, 15 × 15cm^2^, and 30 × 30 cm^2^ are 0.5%, 4.6%, and 5.6% for open field and 0.9%, 4.7%, and 7.2% for motorized 60° wedge field when FSD varies from 80 cm to 120 cm. The build-up curve for 5 × 5 cm^2^, 15 ×15 cm^2^, and 30 × 30 cm^2^ field sizes with a 1.0-cm Perspex blocking tray at 80 cm, 100 cm, and 120 cm FSD are shown in Figure 2c. The maximum PDD difference seen at the surface (PDD_0_) for 5 × 5 cm^2^, 15 × 15 cm^2^, and 30 × 30 cm^2^ fields are 5.6%, 22.8%, and 29.6%, respectively, as FSD varies from 80 cm to 120 cm. Figure 2d shows the comparison of build-up doses for open fields *vs*. motorized 60° wedge and block tray fields at 100 cm FSD.

**Figure 2a:**
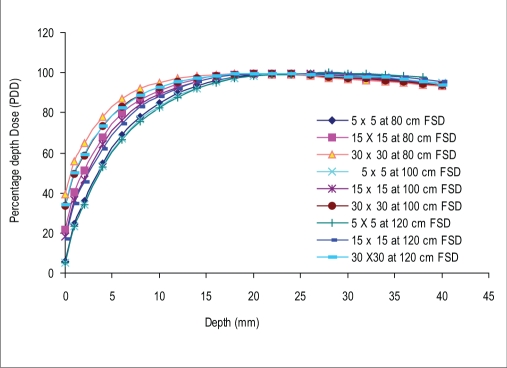
Comparision of build-up doses for open fields at 100 cm FSD vs. 80 cm and 120 cm FSD for the various field sizes

**Figure 2b:**
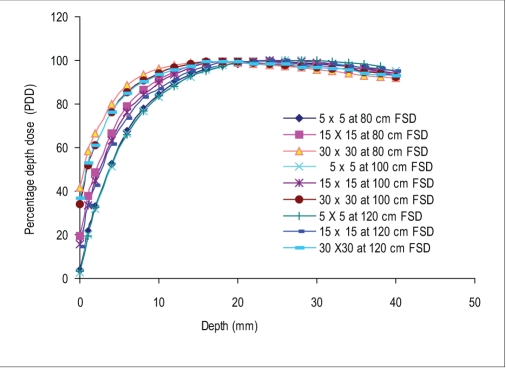
Comparision of build-up dose for motorized wedge at 100 cm FSD vs 80 cm and 120 cm FSD for the various field sizes

**Figure 2c:**
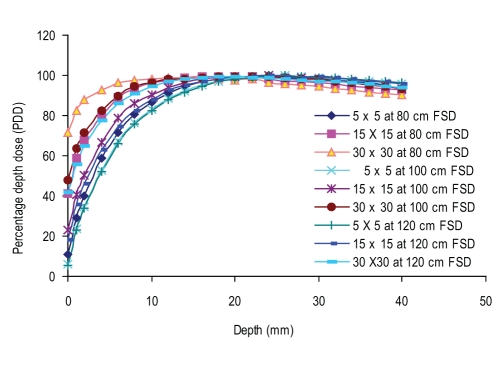
Comparision of build-up dose for tray fields at 100 cm FSD vs 80 cm and 120 cm FSD for the various field sizes

**Figure 2d:**
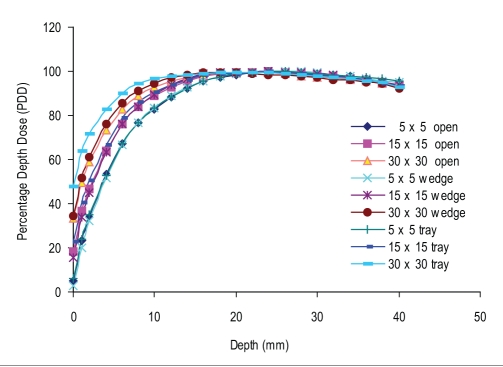
Comparision of build-up doses for open fields vs tray and wedge fields at 100 cm FSD

## Discussion

Megavoltage X-rays are used for the treatment of deep-seated tumors due to its skin-sparing effect. There is a strong relation between the field size and surface to build-up dose. The surface dose increases as field size increases from 3 × 3 cm^2^ to 30 × 30 cm^2^ (3.45 % to 39.06%, 3.2% to 34.47%, and 3.15 % to 33.4% at 80 cm, 100 cm, and 120 cm FSD, respectively) [Table 1]. The percentage depth dose at surface (PDD_0_) increased almost linearly with field size (~1.124%/cm, ~1.643%/cm, and ~1.20%/cm) for open, tray, and wedge field, respectively, at 100 cm FSD. Our results agree with the literature; for example, the surface dose measured by Klein *et al*.[9] increased nearly linearly with F.S (1.16%/cm) for open fields. The increase in surface dose is due to the flattening filter, monitor chambers, collimators, and distance to phantom surface.[10,11] The PDD_0_ at 80 cm, 100 cm, and 120 cm FSDs were 13.89%, 11.71%, and 10.74 %, respectively, for a 10 × 10 cm^2^ open field. When the blocking tray was introduced, the PDD_0_ was increased to 26.29%, 14.01%, and 11.53% for the same field and same FSD. These results agree with reports in the literature; for example, the surface dose measured by Nadir *et al*.[12] was 12.5% and 13.2% for 10 × 10 cm^2^ open and tray fields, respectively, at 100 cm FSD. PDD_0_ values with the acrylic block tray were higher than those with open fields. This effect was more prominent with larger field sizes and smaller FSD. For example, Table 1 shows similarity in surface dose (PDD_0_) with open field in comparison with that with an acrylic block tray in place for 5 × 5 cm^2^ field size at 100 cm FSD; however, the surface dose changed from 34.47% to 47.69% at 100 cm FSD and 39.06% to 71.33% at 80 cm FSD by adding an acrylic block tray for 30 × 30 cm^2^ field size. Our measured results agree with data published in the literature ;for example, PDD_0_ for 20 × 20 cm^2^ open field was 24.94%, Mellenberg[6] measured skin dose value for 20 × 20 cm^2^ field was 23.4% for 15 MV photons. The tray eliminates the electrons from upstream and generates new secondary electrons by itself.[13] The number of electrons originating at the tray is larger (as Lucite generates more electrons due to interactions[14] than the number of electrons eliminated by the tray); secondary electrons originating at the tray can penetrate the tray and reach the patient. It may be concluded that the effects of the blocking tray on the surface dose were quite significant and increased with increasing field size.

When motorized 60° wedge was introduced, the PDD_0_ was slightly decreased to 11.32%, 9.74%, and 8.9% at 80 cm, 100 cm, and 120 cm, respectively, for 10 × 10 cm^2^ field. These results agree with the literature; for example, the surface dose measured by Nadir *et al*.[12] was 12.5% and 13.2% for 10 × 10 cm^2^ open and tray fields, respectively, at 100 cm FSD for 15 MV. According to Li *et al*.[13] the skin doses for 30° wedge field was 10.4% and 10.2% for 8 MV and 18 MV of 10 × 10 cm^2^ field at 100 cm FSD. PDD_0_ for motorized 60° wedge field increased as field size increased, but PDD_0_ values for 60° MW fields were lower than with the same open fields up to 20 × 20 cm^2^ field for all the FSDs. Kim *et al*.[15] reported that physical wedge eliminates electrons from upstream and also generates electrons itself. They noted that the number of electrons produced in the wedge is less than the number of electrons eliminated by the wedge for smaller field sizes and smaller wedge angles. According to their report, this effect is reversed only with larger field sizes and larger wedge angles. Our data agrees with this report (for example, surface dose measured for 30 × 30 cm^2^ open field were 39.06%, 34.47%, and 33.4% at 80 cm, 100 cm and 120 cm FSD, and for motorized 60° wedge it was 41.37%, 36.82%, and 34.2% at 80 cm, 100 cm, and 120 cm, respectively).

The percentage depth dose at the surface (PDD_0_) decreased rapidly with increasing FSD for tray field, but for the open and wedge field it decreased slightly with increasing FSD. Figures 2a and 2b shows the build-up curves for open and wedge fields at various FSDs. The maximum PDD difference seen at surface for 5 × 5 cm^2^, 15 × 15 cm^2^, and 30 × 30 cm^2^ were 0.5%, 4.6%, and 5.6% for open field and 0.9%, 4.7%, and 7.2% for motorized wedge field, respectively, as FSD varied from 80 to 120 cm, and reducing with depth. This is due to the field size is still quoted at the isocenter (collimator positions remain unchanged) would explain the closeness of the build-up doses measured. The area inside the treatment head of the accelerator, which produces and allows electron contamination to escape, remains constant as the FSD varied. Electrons produced within the head of accelerator are relatively high energy; when these electrons are required to travel say 20 cm more or 20 cm less in air it will not significantly change their range in the phantom by a sizeable amount.

The surface to build-up curve for 5 × 5 cm^2^, 15 × 15 cm^2^ and 30 × 30 cm^2^ field sizes with 1.0 cm Perspex blocking tray at 80 cm, 100 cm, and 120 cm FSD are shown in Figure 2c. The maximum PDD difference seen at the surface for 5 × 5 cm^2^, 15 × 15 cm^2^, and 30 × 30 cm^2^ are 5.6%, 22.8%, and 29.6%, respectively, as FSD varies from 80 to 120 cm, and the difference decreases with depth in the build-up region. These results point towards the measurable difference in low-energy electron contamination produced by the Perspex blocking tray being recorded at different FSDs, as these electrons have a large angular distribution, it is hypothesized that their dose distribution decreases quite considerably with increasing FSD.

Figure 2d shows a comparison of build-up doses for open field *vs*. tray field and MW field at100 cm FSD. There is no significant difference seen in the build region for the wedge fields. A similarity in surface and build-up dose with open field, in comparison with an acrylic block tray in place for 5 × 5 cm^2^ field size at 100 cm FSD, but surface dose (PDD_0_) changed from 34.47% to 47.69% at 100 cm by adding an acrylic block tray for 30 × 30 cm^2^ field size.

The clinical significance of these results is that for open and wedge fields there is no significant change in dose delivered to the skin and subcutaneous tissue with isocentric or extended treatments. However, with the use of block trays, the effect of FSD changes the dose delivered to this region. Increase of skin dose can cause early radiation effects such as erythema or late radiation-induced effects such as hypoxia, fibrosis, etc. We reported measurements performed at 80 cm FSD, because FSDs less than 100 cm may be encountered during isocentric treatments and surface dose estimates could be obtained by interpolation between 80 cm and 100 cm. Treatment planning systems do not provide correct estimates of surface doses, and this study will therefore add some data in this area for the Elekta machines of present design.

## Conclusions

The surface dose increases with field size for open fields. PDD_0_ for MW field increased as field size increased, but PDD_0_ values for MW fields were lower than for the same open fields. MW eliminates secondary electrons but generates new electrons. PDD_0_ values with the acrylic block tray were higher than those with open field. The effects of the blocking tray on the surface dose were quite significant and increased with increasing field size. Changes in FSD produces only a minimal effect on the dose for open and motorized wedge field beams; however, a significant effect is seen for blocking trays. The dose in the build-up region increases with decreasing FSD for fields with blocking trays due to the influence of electron contamination produced by the Perspex blocking tray.
